# Effect of Light Conditions on Polyphenol Production in Transformed Shoot Culture of *Salvia bulleyana* Diels

**DOI:** 10.3390/molecules28124603

**Published:** 2023-06-07

**Authors:** Marta Krzemińska, Katarzyna Hnatuszko-Konka, Izabela Weremczuk-Jeżyna, Aleksandra Owczarek-Januszkiewicz, Wiktoria Ejsmont, Monika A. Olszewska, Izabela Grzegorczyk-Karolak

**Affiliations:** 1 Department of Biology and Pharmaceutical Botany, Medical University of Lodz, Muszynskiego 1, 90-151 Lodz, Poland; marta.wojciechowska2@stud.umed.lodz.pl (M.K.); izabela.weremczuk-jezyna@umed.lodz.pl (I.W.-J.); wiktoria.ejsmont@stud.umed.lodz.pl (W.E.); izabela.grzegorczyk@umed.lodz.pl (I.G.-K.); 2 Department of Molecular Biotechnology and Genetics, Faculty of Biology and Environmental Protection, University of Lodz, Banacha 12/16, 90-237 Lodz, Poland; katarzyna.hnatuszko@biol.uni.lodz.pl; 3 Department of Pharmacognosy, Medical University of Lodz, Muszynskiego 1, 90-151 Lodz, Poland; aleksandra.owczarek@umed.lodz.pl

**Keywords:** *Agrobacterium rhizogenes*, LEDs, light treatment, transformed shoots, rosmarinic acid, *Salvia bulleyana*, salvianolic acid K

## Abstract

Various strategies have been used to increase the efficiency of secondary metabolite production in *Salvia* plants. This report is the first to examine the spontaneous development of *Salvia bulleyana* shoots transformed by *Agrobacterium rhizogenes* on hairy roots and the influence of light conditions on the phytochemical profile of this shoot culture. The transformed shoots were cultivated on solid MS medium with 0.1 mg/L of IAA (indole-3-acetic acid) and 1 mg/L of m-Top (meta-topolin), and their transgenic characteristic was confirmed by PCR-based detection of the *rol*B and *rol*C genes in the target plant genome. This study assessed the phytochemical, morphological, and physiological responses of the shoot culture under stimulation by light-emitting diodes (LEDs) with different wavelengths (white, WL; blue, B; red, RL; and red/blue, ML) and under fluorescent lamps (FL, control). Eleven polyphenols identified as phenolic acids and their derivatives were detected via *ultrahigh-performance liquid* chromatography with diode-array detection coupled to electrospray ionization tandem mass spectrometry (UPLC-DAD/ESI-MS) in the plant material, and their content was determined using high-performance liquid chromatography (HPLC). Rosmarinic acid was the predominant compound in the analyzed extracts. The mixed red and blue LEDs gave the highest levels of polyphenol and rosmarinic acid accumulation (respectively, 24.3 mg/g of DW and 20.0 mg/g of DW), reaching two times greater concentrations of polyphenols and three times greater rosmarinic acid levels compared to the aerial parts of two-year-old intact plants. Similar to WL, ML also stimulated regeneration ability and biomass accumulation effectively. However, the highest total photosynthetic pigment production (1.13 mg/g of DW for total chlorophyll and 0.231 mg/g of DW for carotenoids) was found in the shoots cultivated under RL followed by BL, while the culture exposed to BL was characterized as having the highest antioxidant enzyme activities.

## 1. Introduction

The development of civilization diseases, the need to place more emphasis on prevention, and the increase in the resistance of microorganisms to available synthetic drugs are some of the factors that have spurred interest in medicinal plant-derived natural products. The members of the *Salvia* genus are well known for having therapeutic potential, and there is, hence, great interest in increasing the efficiency of the production of their secondary metabolites. Alternative methods for obtaining valuable plant materials without degrading the environment can be offered via plant biotechnology. One such approach involves transformation by *Agrobacterium rhizogenes*, which involves the integration of T-DNA of the Ri plasmid of bacteria into the plant genome. Such transgenic roots are known to be stable and rapid-growing, and they can take part in spontaneous or growth regulator-induced regeneration of adventitious buds for initiating shoot culture [1,2]. However, while several *Salvia* species have been reported to develop hairy roots [3,4,5,6,7], only *S. miltiorrhiza* Bunge has been found to develop transformed shoots on hairy roots [8]. As plants regenerated from hairy roots are non-chimera and are usually genetically stable, they can also be a valuable source of medicinal plants [9]. Moreover, like hairy root cultures themselves, shoot transformants can also produce high levels of secondary metabolites [10,11]. It would, hence, be desirable to establish transgenic shoot cultures for other species of sage.

One interesting candidate with high therapeutic potential is *Salvia bulleyana* [12]. In local Chinese medicine, its roots are used as a substitute for Danshen (roots of *S. miltiorrhiza*). It has been used in cardiovascular diseases, including angina pectoris, and as a treatment for hepatitis and cirrhosis [13,14]. In folk medicine, this raw material has been recommended for kidney diseases, bloody vomiting, pain relief, and painful menstruation. It has also been used as a hypnotic, sedative, and muscle-relaxing agent [13,14]. Like the original Danshen, the therapeutic potential of its raw material seems to be related to the presence of two main groups of secondary metabolites, polyphenols and tanshinones with rosmarinic acid being the dominant component of *S. bulleyana* [15]. However, the limited availability of raw materials from natural sites prevents any detailed analysis regarding its therapeutic potential or use. One alternative way of obtaining this material could be by using in vitro cultures. 

Earlier studies found that *S. bulleyana* hairy root cultures optimized via biotechnological methods produce a high amount of polyphenolic compounds and are competitive with soil-grown plant materials [16,17]. Therefore, the possibility of analyzing spontaneous shoot culture originating from the hairy roots of *S. bulleyana* also seems very attractive. On the other hand, some drawbacks have sometimes been noted for transformed shoot cultures. A comparative study found that transformed plants differ from non-transformed plants in their morphological and physiological characteristics, including having wrinkled leaves, elongation disorders, and dwarf phenotype [18,19]. However, this can be rectified by various methods, such as altering the light conditions. It has been shown that light (type of light source, light wavelength, its intensity, and exposure time) can be an important factor in stimulating plant growth, with some studies suggesting light to be one of the most important factors influencing the biosynthesis of bioactive compounds and, thus, the biological activity of raw materials [20,21,22,23,24]. Secondary metabolites, particularly polyphenols, together with antioxidant enzymes, such as superoxide dismutase (SOD), peroxidase (POD), and catalase (CAT), are scavengers of free radicals and can provide photoprotection against light stress. Therefore, their production can be strongly stimulated under light-stress conditions.

The present report is the first to describe the spontaneous regeneration of transformed shoots of *S. bulleyana* from a hairy root culture. The aim of the study was to estimate the potential of the obtained culture to produce bioactive compounds. After confirming the transformation at the molecular level, the culture growth conditions were optimized in order to achieve high production of bioactive polyphenolic compounds, particularly valuable rosmarinic acid, and culture biomass accumulation based on the light environment. Additionally, the quantities of photosynthetic pigments (chlorophylls a and b, and carotenoids) and the activity of antioxidant enzymes (POD, CAT, and SOD) were estimated from the shoot extracts of each light treatment using spectrophotometry.

## 2. Results

*Salvia bulleyana* hairy roots were obtained from shoots transformed by wild-type *A. rhizogenes* A4 strain [17]. Two to three weeks after infection, the roots that appeared at the infection sites were cut off and cultured in a liquid WP medium with ampicillin to eliminate bacteria. At the time, spontaneous shoot formation was observed from passages 4 to 6 on a single clone. The frequency of spontaneous shoot regeneration was low (only two or three shoots appeared on the hairy root culture). The obtained shoot culture (P7 clone) was used for further analysis, which is described in the following sections.

### 2.1. Molecular Analysis

**Figure 1:**
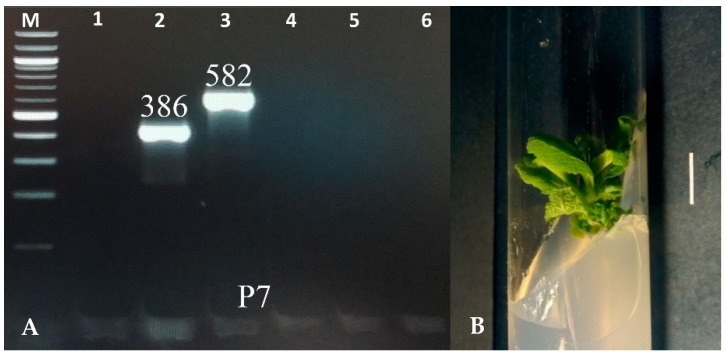
PCR-amplified products isolated from the genomic DNA of transformed shoots of *S. bulleyana* clone P7 (**A**). Lane: M—marker DNA of 100–1000 bp; 1—*vir*G (319 bp); 2—*rol*B (386 bp); 3—*rol*C (582 bp); 4—*rol*D (204 bp); 5—*aux*1 (500 bp); and 6—*aux*2 (774 bp). Transformed shoot culture of *S. bulleyana* clone P7 grown on MS agar medium with 0.1 mg/L of IAA and 1 mg/L of m-Top (passage 4). The scale bar is 1 cm (**B**).

The transgenic characteristic of the shoots was confirmed using the PCR method with primers for the *aux*1, *aux*2, *rol*B, *rol*C, and *rol*D genes. DNA was extracted using the CTAB method [25]. The fragments corresponding to the *rol*B (386 bp) and *rol*C (586 bp) genes were identified in the shoots of the P7 clone. The *vir*G gene, a characteristic of bacteria, was not noted in the plant material, indicating effective elimination of bacteria from the culture (Figure 1). None of the amplified genes was detected in the control shoots, which grew in vitro from *S. bulleyana* seeds.

### 2.2. Secondary Metabolite Content

The UPLC-PDA-ESI-MS analysis of the extracts from the *S. bulleyana* shoot cultures showed an identification of 11 compounds representing phenolic acid derivatives; these had previously been detected in untransformed shoots [26]. The constituents were analyzed in the negative ion mode and identified based on the retention time, and the PDA and MS spectra were compared with the reference standards or data from previous studies [17,26]. The detailed fragmentation data for individual compounds are presented in a previous paper [26].

**Figure 2:**
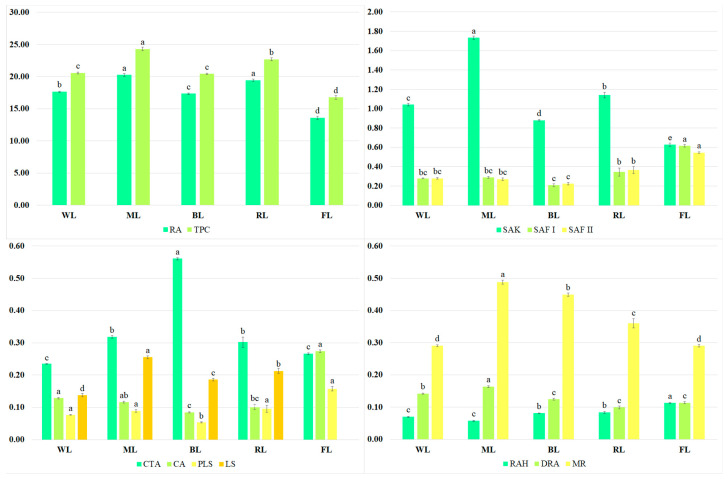
Contents (mg/g of DW) of TPC, RA, SAK, SAF I and II, CTA, CA, PLS, LS, RAH, DRA, and MR in the transformed shoots of S. *bulleyana* grown for five weeks on MS solid medium with 0.1 mg/L of IAA and 1 mg/L of m-Top under different light conditions. Values marked with the same letter do not show statistical differences at the significance level of *p* < 0.05.

The predominant metabolite present in the extracts was rosmarinic acid (Figure 2). Besides RA, a few of its derivatives, such as rosmarinic acid hexoside (RAH), dehydrorosmarinic acid (DRA), and methyl rosmarinate (MR), were present in the *S. bulleyana* shoot culture. Other bioactive metabolites detected in the analyzed extracts were caffeic acid (CA), caffeoyl-threonic acid derivative (CTA), protolithospermic acid isomer (PLS), lithospermic acid isomer (LS), salvianolic acids such as salvianolic acid K (SAK), and salvianolic acid F isomers I and II (SAF I and SAF II).

The total phenol content (TPC) in the extracts ranged from 20.4 to 24.3 mg/g of dry weight (DW) (Figure 2), depending on the LED treatment; these values were 22–45% higher than those obtained from the shoots grown under the control conditions (under light emitted by fluorescent lamps). The highest RA levels were recorded for the shoots exposed to ML and RL (approx. 20 mg/g DW); their levels were approx. 15% higher than those obtained for other LED conditions and approx. 50% higher than for the control (Figure 2). ML also stimulated the production of SAK (1.73 mg/g of DW), MR (0.488 mg/g of DW), DRA (0.164 mg/g of DW), and LA isomer (0.255 mg/g of DW). However, FL was the most effective for CA accumulation (0.274 mg/g of DW), and it was two times higher than under the best LED treatment, i.e., WL. Under the control conditions, the highest amounts of PLS (0.158 mg/g of DW, RAH (0.113 mg/g of DW), and SAF isomers (0.615 and 0.545 mg/g of DW, respectively) were also observed (Figure 2). In most cases, the lowest production of active compounds was noted under BL.

### 2.3. Growth and Morphological Characterization of the Culture

**Figure 3:**
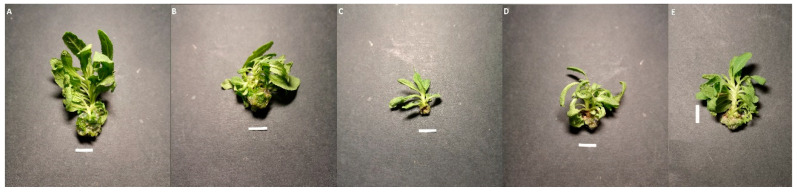
Transformed shoots of *S. bulleyana* after 5 weeks of growth on a solid MS medium with 0.1 mg/L of IAA and 1 mg/L of m-Top under various LED lighting conditions: (**A**)—WL, (**B**)—ML, (**C**)—BL, (**D**)—RL, and (**E**)—FL. The scale bar is 1 cm.

The *S. bulleyana* shoot culture, which transformation was confirmed by the presence of the *rol*B and *rol*C genes, was grown on MS medium with 0.1 mg/L of IAA and 1 mg/L of m-Top. The types and concentrations of plant growth regulators were chosen based on previous experiments with untransformed shoot culture [26]. The transformed shoots looked similar to the untransformed ones grown under the same conditions. However, some phenotypical changes were observed (Figure 3), such as short internodes, limited apical dominance, stunted growth, and ease of rooting. The culture also displayed worse proliferation potential than untransformed *S. bulleyana* shoots cultivated in vitro [26].

Two main criteria should be considered for the successful production of secondary metabolites in in vitro culture: high yield of secondary metabolites in plants and effective growth of culture and its stress resistance under conditions where stress factors are used to stimulate the production of bioactive compounds. In order to optimize growth, the transformed *S. bulleyana* shoots were grown for five weeks under different LED lighting conditions. The control consisted of transgenic shoots grown under light emitted by fluorescent lamps (FL). The obtained results indicate that the length of the light wave affects shoot growth, morphology, and proliferation (Figure 3, Table 1).

The shoots exposed to white and mixed light had the highest shoot multiplication rate, with a range of 2.72–3.04 (Table 1). A similar level of proliferation was noted for the control shoots, but the percentage of produced shoots in comparison to buds was 20–26% for LED light and only 5% for fluorescent light (Table 1).

**Figure 4:**
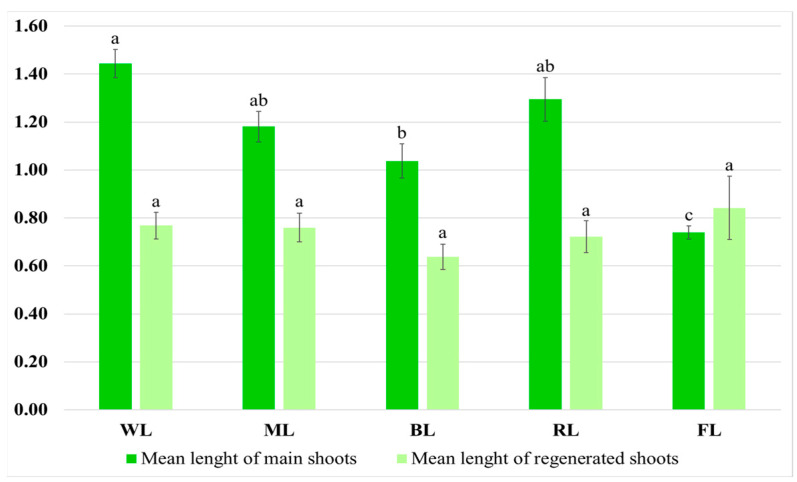
Mean length (cm) of main shoots and regenerated shoots after five weeks of cultivation on solid MS medium with 0.1 mg/L of IAA and 1 mg/L of m-Top under different light conditions. Values marked with the same letter do not show significant differences at *p* < 0.05.

The light conditions also influenced shoot morphology. The shoots grown under WL had an intense, dark green color and numerous short-petioled leaves with the largest laminas. The greatest mean length of the main shoot was observed under this condition, i.e., 1.44 cm (Figure 4).

The WL culture also demonstrated a high multiplication ratio and intensive growth, resulting in the greatest FW and DW, i.e., 1.22 g of FW and 0.145 g of DW, and, thus, showing an almost 40-fold increase in biomass during the five-week period. These results were about 25% higher than those observed for the controls and more than double those for the shoots under BL, which was the least favorable condition. Similar growth as under WL was observed for the ML treatment (no significant differences), although the shoots grown under ML had leaves with slightly longer and narrower laminas than those grown under WL, and a brighter color (Table 1, Figure 3).

It was found that selective red or blue light limited culture growth. These conditions also affected the morphology of the cultures. Exposure to RL caused the leaves to be long-petioled and narrow, with the edges of the lamina being folded (Figure 3). However, the main shoot and the newly formed ones did not differ significantly in size from those observed under WL (Figure 3). Although exposure to RL resulted in a decreased proliferation factor, the culture demonstrated the highest ratio of regenerated shoots to buds. The shoots exposed to BL had the lowest biomass (Table 1). They were also the shortest (1.04 cm) (Figure 4). They had the smallest number of leaves, which leaf blade was wider than in the other research samples. In addition, roots occasionally appeared spontaneously on the explants.

Stimulation of the biosynthesis of photosynthetic pigments can be a factor affecting plant growth; therefore, in this study, the contents of photosynthetic pigments (chlorophylls a and b, and various carotenoids) in the *S. bulleyana* shoots cultivated under different LEDs were also determined. The control consisted of transgenic shoots grown under light emitted by fluorescent lamps (FL).

Light wavelength had a significant effect on the production of photosynthetic pigments in the transgenic *S. bulleyana* shoots. The highest level of photosynthetic pigments (1.36 mg/g of DW) was found in the shoots cultivated under RL (Table 2). The total amount of chlorophyll was 1.13 mg/g of DW, and the carotenoid content was 0.231 mg/g of DW, i.e., twice as high as those obtained under FL. RL had a strong effect on the production of chlorophyll b (0.604 mg/g of DW), which level increased 2.5- to 5.5-fold compared to others. BL also strongly influenced the production of photosynthetic pigments. All LED treatments achieved a higher total chlorophyll content compared to FL. In the case of carotenoids, their lowest levels were recorded in the control shoots, as well as those under ML.

### 2.4. Activities of Antioxidant Enzymes in Culture

To assess the effect of light stress on the culture, after five weeks of cultivation under different lighting conditions, the activities of the antioxidant enzymes, POD, SOD, and CAT, were also assessed in the transformed shoots of *S. bulleyana*.

All three antioxidant enzymes demonstrated a higher activity in all LED treatments compared to the control. The greatest activities were observed under BL: 28.56 U/mg of protein (POD), 7.15 U/mg of protein (SOD), and 8.60 U/mg of protein (CAT) (Table 3). The lowest activities of all antioxidant enzymes were recorded for the FL culture (Table 3): 10 times lower for SOD, 20 times lower for POD, and almost 40 times lower for CAT compared to BL.

## 3. Discussion

The aim of the present research was to increase the efficiency of *S. bulleyana* transformed shoot culture. The culture used in the present study was regenerated spontaneously from hairy roots created by *A. rhizogenes* strain A4 transformation. It is the first report on obtaining transgenic shoots from this plant. Previously, among *Salvia* species, *S. miltiorrhiza* Bunge has been found to develop transformed shoots on hairy roots, but in this case, shoot regeneration was stimulated by the presence of growth regulators in the growth medium [8]. On the other hand, the spontaneous formation of shoots on hairy roots has been reported for some other medicinal plants [27,28,29].

Plant biotechnology offers the possibility to increase the biosynthesis of bioactive compounds through different strategies, such as by selecting highly productive lines or via the optimization of growth conditions (composition of the growth medium, temperature, and light conditions used) [30]. Light is one of the most important abiotic factors affecting plant growth, morphogenesis, and metabolism. Previously, in vitro plant cultures have typically used fluorescent lamps; however, recent studies have demonstrated increasing interest in LEDs because of their wavelength specificity. LED technology has many advantages; it provides lower heat emission and variable light intensity and wavelength. Additionally, LED lamps have a longer life and occupy a smaller area than conventional white fluorescent light [31]. Previous studies have indicated that selected LEDs can promote secondary metabolite production, biomass accumulation, and plant elongation in some species [32]. However, different plant species exhibit different responses to a given set of lighting conditions.

Light is necessary for the life of organisms, but it can also be a stress factor that generates the production of reactive oxygen species (ROS) and stimulates the organisms’ defense responses. As a result, plants can increase the production of specific secondary metabolites to accommodate themselves to stressful conditions. For example, polyphenols can absorb radiation and act as a barrier against a high light intensity and participate in the neutralization of ROS [33]. As expected, the light conditions also affected the accumulation of polyphenolic compounds in the transformed shoots of *S. bulleyana* in the present study, although the same polyphenols were detected in the shoots from all light treatments.

The most valuable bioactive compound present in the extracts was rosmarinic acid. This ester of caffeic acid and 3,4-dihydroxyphenyllactic acid has numerous biological and pharmacological activities [34,35]. Previous studies have confirmed its anti-inflammatory, antiviral, and antibacterial activities. Rosmarinic acid, being a potent free radical scavenger, is a precious neuroprotective, cardioprotective, and hepatoprotective agent. It also shows a cognitive-enhancing effect via the inhibition of the prolyl oligopeptidase and amyloid-β aggregation pathways. Long-term exposure to RA in diet can be beneficial for cancer chemoprevention, and RA has also demonstrated anticancer potential. Previous studies on various cancer cell lines have shown that RA increases the expression of pro-apoptotic genes, inhibits the activity of anti-apoptotic proteins, inhibits cancer cell proliferation by arresting the cell cycle, and reduces metastatic capacity [34,35].

Every LED treatment was characterized by a higher total polyphenol content (20.4–24.3 mg/g of DW) and rosmarinic acid level (17.3–20.3 mg/g of DW) than the shoots grown under FL (16.8 and 13.6 mg/g of DW, respectively). The maximum TPC, rosmarinic acid, and SAK production, as well as that of several other polyphenols, was achieved under the mixed light conditions. The content of RA obtained in these conditions was three times higher than in the shoots of two-year-old intact plants, and the content of TPC was double [15]. The combination of blue and red light also significantly improved the overall flavonoid content of *Anoectochilus roxburghii* (Wall.) Lindl. culture [36]. Similarly, ML yielded the greatest production of chlorogenic acid in *Dracocephalum forrestii* W. W. Smith shoot culture and callus *Peucedanum japonicum* Thunb. [37,38]; in contrast, the production of chlorogenic acid decreased under RL alone. Similar results were reported for *Ruta graveolens* L.: red light inhibited chlorogenic acid biosynthesis compared to other LED treatments [39]. Among all LED treatments, blue light turned out to be the least favorable for the production of metabolites in the *S. bulleyana* shoots; this was also observed in *Ocimum basilicum* L. in which the lowest RA concentration was noted under BL [40]. However, some studies have indicated that BL as a stressor stimulates the production of polyphenolic compounds in inter alia cultures of *Rehmania glutinosa*, *Aronia melanocarpa* (Michx.) Robertson & Phipps, *A. arbutifolia* (L.) Pers, *A. prunifolia* (Marshall) Rehder, or *Prunella vulgaris* L. [41,42,43]. BL is high-energy radiation, and although it can stimulate secondary metabolism due to stress reactions, it can also negatively impact the growth of sensitive species [44].

Since a high content of ROS results in changes in the activity of major plant antioxidant enzymes (POD, SOD, and CAT) associated with defensive functions [45,46,47], the level of stress under different light conditions in the present study was estimated by determining their activity in the plant material. In the case of the *S. bulleyana* culture under BL, strong oxidative stress was noted as indicated by a dramatic increase in the activity of all antioxidant enzymes. Similarly, BL was also found to have the strongest influence on antioxidant enzyme activity in *R. glutinosa* and *Oncidium* [41,48]. In addition, all LEDs used had a great stimulatory effect on the activity of the tested antioxidant enzymes in the *S. bulleyana* shoots compared to FL; a similar response has previously been observed for other species [37,41].

Meanwhile, the high sensitivity of a plant to stress conditions may be manifested in the inhibition of its growth, while the growth of the culture is as important a parameter of high productivity as the content of bioactive metabolites in the plant material. It is all the more important that the transgenic nature of the culture itself may predispose to growth disorders and specific morphological features. While transformed shoots demonstrate increased rooting and root mass, they may suffer less development in the above-ground part; transformed shoots can also be characterized by wrinkled leaves and inhibited elongation of internodes, and may be stunted [18]. *S. bulleyana* transformants also showed some variations in phenotype, including abnormal stem growth and short internodes. Mehrotra et al. (2013) linked such morphological changes to the incorporation of *A. rhizogenes* gene’s plasmid T-DNA into the plant genome [49]. Some reports suggest that an overexpression of the *rol*C gene could be responsible for the spontaneous formation of adventitious shoots and some morphological anomalies by influencing gibberellin metabolism [50,51,52]. Many *rol*C transformants, including *Pelargonium* and *Chrysanthemum morifolium* Ramat, have been characterized as having a dwarf phenotype with reduced apical dominance, variation in leaf size and leaf number, and increased rooting ability [53,54]. Fragments corresponding to the *rol*C gene, together with the *rol*B gene, were also found to be present in the genome of the *S. bulleyana* shoots.

Therefore, in this study, we used different lighting conditions to stimulate the production of polyphenolic compounds and to also improve the growth of transformed shoots. As it has been reported, the use of monochromatic light sources during cultivation might be effective and economically justified because they do not include wavelengths irrelevant to plant development and production [55]. A relatively large number of studies have reported that BL has the high potential to influence not only production but also the growth parameters of farmed species. For example, blue diodes turned out to be the most effective sources of light for the cultivation of the shoots of *Dracocephalum forrestii* [56], *Anoectochilus roxburghii* [47], and *Rehmania glutinosa* [41]. However, blue or red light alone significantly limited the growth of the *S. bulleyana* transformed shoots. In this case, the highest multiplication factor and biomass accumulation were obtained under WL and under ML, which indicates that the culture needs at least two wavelengths to grow effectively, viz. red and blue. Similarly, *Carpesium triste* Maxim. biomass increased more under ML than BL and RL alone [57]. Exposure to mixed light was also particularly beneficial for the biomass accumulation of *Dendranthema grandiflorum* (Ramat.) Kitam. [55].

A parameter sensitive to light conditions that can be an indicator of growth and metabolism in plants is the level of photosynthetic pigments. Essentially, chlorophyll has two absorption peaks, viz. at wavelengths of 430–450 nm and 640–660 nm; as such, it is possible that blue (420–500 nm) light and red (640–700 nm) light together affect the formation and metabolism of chlorophyll in plants [58]. Since treatment with both of these wavelengths was necessary to stimulate growth and production in the shoots of *S. bulleyana*, this study also estimated the effect of light conditions on the contents of photosynthetic pigments, including chlorophyll in the culture.

The highest levels of photosynthetic pigments in the transformed shoots of *S. bulleyana* were recorded under RL; chlorophyll and carotenoid levels were twice as high when grown under red LED conditions than under control conditions. Slightly lower levels of chlorophyll and similar carotenoid levels were found in the shoots treated with BL. Exposure to RL also stimulated the production of chlorophyll in the shoots of *Rehmania glutinosa* [41], whereas for *Dracocephalum forrestii* transformed shoots, the most favorable treatments turned out to be those using BL [56]. However, the high levels of photosynthetic pigments in the *S. bulleyana* transformants under blue or red LED alone favored increased photosynthesis but did not stimulate growth. This could be due to the low importance of the photosynthesis process for in vitro cultures, which can obtain sugar from the medium; this can distinguish the response obtained under culture conditions from that associated with greenhouse cultivation.

## 4. Materials and Methods

### 4.1. Plant Materials

Transformed root cultures of *S. bulleyana* were established as described previously [17]. A shoot culture was obtained via the spontaneous regeneration of transformed roots (C7 root clones) (P7). These cultures were grown on MS medium with solidified agar (0.7%) and supplemented with 0.1 mg/L of IAA (indole-3-acetic acid) and 1 mg/L of m-Top (meta-topolin). The shoots were cut from the roots and cultivated in a growth chamber at a temperature of 26 ± 2 °C, humidity of 80–90%, and illumination with fluorescent lamps (PPFD 40 μM/m^2^∙s) with the use of a light/dark photoperiod (16/8 h).

### 4.2. Polymerase Chain Reaction Analysis

The genetic transformation of the transformed shoots (P7) of *S. bulleyana* was confirmed using Polymerase Chain Reaction (PCR). A culture of unaltered shoots obtained from sterilized seeds was used as a negative control. Genomic DNA was extracted from the shoot culture following the CTAB protocol as described earlier [25]. The plant samples were frozen using liquid nitrogen and ground into a powder. Then, the CTAB buffer, based on the cationic detergent cetyltrimethylammonium bromide, was added to facilitate cell lysis. DNA was separated from other molecules via phenol/chloroform extraction, followed by recovery through precipitation with isopropanol. The obtained DNA was used as a template for amplification. The PCR was performed with the following primers: 5′-GCT CTT GCA GTG CTA GAT TT-3′ (forward primer) and 3′-CT CTC CAT CGA ACG TGG AAG-5′ (reverse primer) to amplify a 386 bp fragment of the *rol*B gene; 5′-CTC CTG ACA TCA AAC TCG TC-3′ (forward primer) and 3′-AC ATG GGT ATT GAG CTT CGT-5′ (reverse primer) to amplify a 582 bp fragment of the *rol*C gene; 5′-GAT GAT TTT CGT TTT ATC AAG-3′ (forward primer) and 3′-C AAA TTC ATA GGA GAC AGG AAG-5′ (reverse primer) to amplify a 204 bp fragment of the *rol*D gene; 5′-ATC TTA GTC ACT TCA TAG CAG TT-3′ (forward primer) and 3′-G AAC AAG AAG ATA GAG TTT TTC-5′ (reverse primer) to amplify a 500 bp fragment of the *aux*1 gene; 5′-ATA TCT GCT TCA ACA AAA GTA AC-3′ (forward primer) and 3′-A TAA TAG CAA AGC TAA TTG AGT-5′ (reverse primer) to amplify a 774 bp fragment of the *aux*2 gene; and 5′-AGT TCA ATC GTG TAC TTT CCT-3′ (forward primer) and 3′-TCT GAC CTG TGA CTT ATA GTC-5′ (reverse primer) to amplify a 319 bp fragment of the *vir*G gene. The reaction was performed using a Termocycler Biometra UNO II (*Biometra*, Göttingen, Niedersachsen, Germany) with a thermal gradient. The reaction products and a standard DNA marker were run on a 2% agarose gel, stained with ethidium bromide (90 V at 90 min), and then photographed using the gel documentation system.

### 4.3. Phytochemical Analysis

The qualitative and quantitative profile of the hydromethanolic extract of the transformed shoot culture of *S. bulleyana* was investigated as described earlier [17,26]. The identification of the detected polyphenols was confirmed by the UPLC-DAD/ESI-MS analysis based on the PDA and MS spectra, as consistent with the literature. The polyphenol content and the sum of polyphenols in the plant materials obtained via different light treatments were analyzed based on the HPLC method using an Elite LaChrom Hitachi system (Merck, Darmstadt, Germany). For the HPLC analysis, an Ascentis Express C-18 column (7.5 cm × 4.6 mm, 2.7 μm; Supelco, Bellefonte, PA, USA) was used with a solvent system containing 0.5% aqueous solution of orthophosphoric acid (A) and acetonitrile (B). The detection was set at 325 nm. The procedure was performed as described by Wojciechowska et al. (2020) [17]. The applied method was validated in terms of linearity, precision, accuracy, and sensitivity according to the guidance of the International Council for Harmonisation [59]. The linearity of the method for the used standards was confirmed in the whole range of analytical concentrations with r > 0.999. The precision (RSD for a peak area < 5%), accuracy (recoveries in the range of 96–103%), and sensitivity (LODs in the range of 1.93–8.13 ng) were also found adequate. The amount of each compound was expressed as mg/g of DW (dry weight) of plant material.

### 4.4. Culture Growth, Morphology, and Physiology under Different Light Conditions

The shoot tips of the transformed *S. bulleyana* shoots of clone P7 with a single node, which were 0.5–1.0 cm long, were grown on solidified agar (0.7%) MS medium containing 0.1 mg/L of IAA and 1 mg/L of m-Top. Different lighting conditions were created using LED lighting: red light (670 nm), blue light (430 nm), white light (390–760 nm) and mixed light (70% red light and 30% blue light) (PMX Sp. zo.o., Niepolomice, Poland). The range of wavelengths emitted by the LED lamps was measured using the BTS256-LED tester. The shoots grown under illumination with fluorescent lamps (PPFD 40 μM/m^2^∙s) were used as a control. 

The shoots were grown in a growth chamber at a temperature of 26 ± 2 °C and a humidity of 80–90% using a 16 h/8 h light/dark photoperiod. After five weeks of growth, the morphology of the culture, the length of the main shoot (cm), the number of regenerated shoots/buds, the length of regenerated shoots (cm), and the fresh weight (FW) and dry weight of the culture (DW) were established. The multiplication factor, expressed as the mean value of the sum of developed shoots and buds per explant, was also calculated.

The chlorophyll and carotenoid contents were determined according to Weremczuk-Jeżyna et al. (2021) [56]. Briefly, the samples were macerated using cool (4 °C) 80% acetone. The pigment content was established using a Ray Leigh UV-1601 spectrophotometer (Beijing Reyleigh Corp., Beijing, China). Absorbance was tested at the following wavelengths: 664 nm for chlorophyll a, 647 nm for chlorophyll b, and 470 nm for carotenoids. The levels of pigments were expressed as mg/g FW. 

The shoots grown under LED lighting were also studied for the activities of the antioxidant enzymes CAT, POD, and SOD, as described by Weremczuk-Jeżyna et al. (2021) [56]. The extracts needed for the analysis were obtained by grinding (under 4 °C) fresh biomass of the shoots (0.5 g) with 4 mL of phosphate buffer (pH = 7.5) with the addition of 0.5 mM of EDTA. The supernatant was used to determine CAT, POD, and SOD activities using spectrophotometric assays (a Ray Leigh UV-1601 spectrophotometer, Beijing Reyleigh Corp., Beijing, China). The POD activity was determined based on the increase in absorbance at 470 nm due to guaiacol (Sigma-Aldrich, Darmstadt, Germany) oxidation. The SOD activity was determined as the ability to inhibit the reduction of nitro blue tetrazolinum (NBT) (Sigma-Aldrich, Darmstadt, Germany) and the absorbance of the samples was determined at 560 nm, while CAT activity was measured based on the decrease in H_2_O_2_ (Sigma-Aldrich, Darmstadt, Germany) at 240 nm. The specific enzyme activity was calculated as the units of enzyme activity per mg of protein of the samples (U/mg protein). Protein concentration was determined according to Bradford (1976) [60].

### 4.5. Statistical Analysis

All experiments were performed in triplicate. The results were presented as mean ± standard error. A one-way analysis of variance (one-way ANOVA) was performed to evaluate the significant differences among the treatments, followed by Tukey’s multiple range test (*p* < 0.05). The statistical analysis was carried out using STATISTICA 10.0 software (STATSoft, Krakow, Poland). 

## 5. Conclusions

Our findings provide the first description of transformed shoots of the important medicinal plant *S. bulleyana*. This study demonstrated the effect of different light conditions on transformant growth and estimated their polyphenol biosynthetic abilities. LED light turned out to be more favorable than standard fluorescent light for the production of bioactive compounds in the transformed shoots, with mixed light most strongly stimulating both the accumulation of polyphenolic compounds and the growth of the culture. In these conditions, the total polyphenol content was two times and the level of rosmarinic acid was three times higher than those noted in the above-ground part of two-year-old intact plants. This confirms the effectiveness of the above method to obtain valuable plant material and is a promising introduction to further work on improving the production of polyphenols in *S. bulleyana* culture. On the other hand, the use of selective wavelengths of light, especially blue, caused very strong oxidative stress in the culture, which inhibited growth and limited metabolism, although it increased the biosynthesis of photosynthetic pigments. The poor relationship between the intensity of photosynthesis and the growth of the culture seems to result from the low importance of this process for in vitro cultures, which obtain their carbon source primarily from the growth medium.

## Figures and Tables

**Table 1:** Growth parameters of *S. bulleyana* transformed shoots after five weeks of cultivation on MS medium with 0.1 mg/L of IAA and 1 mg/L of m-Top under different lighting conditions. Values marked with the same letter do not show significant differences (*p* < 0.05).

Light Condition	Fresh Weight (g)	Dry Weight (g)	Proliferation Factor	Ratio of Number of Obtained Shoots to Buds
WL	1.22 ± 0.104 a	0.145 ± 0.010 a	3.04 ± 0.272 a	20:80
ML	1.09 ± 0.107 ab	0.134 ± 0.011 ab	2.72 ± 0.257 ab	26:74
BL	0.545 ± 0.093 c	0.070 ± 0.010 c	1.88 ± 0.254 b	19:81
RL	0.796 ± 0.105 bc	0.100 ± 0.012 bc	2.00 ± 0.267 ab	32:68
FL	0.895 ± 0.048 b	0.109 ± 0.006 b	2.91 ± 0.228 a	5:95

**Table 2:** Effect of light conditions on the contents of photosynthetic pigments (mg/g of DW) in transformed shoots of *S. bulleyana*. Values marked with the same letter do not show statistical differences at the significance level of *p* < 0.05.

Light Condition	Chlorophyll a	Chlorophyll b	Total Chlorophyll	Carotenoids
WL	0.473 ± 0.002 b	0.253 ± 0.003 b	0.723 ± 0.006 c	0.132 ± 0.002 b
ML	0.413 ± 0.004 c	0.211 ± 0.004 c	0.624 ± 0.005 d	0.093 ± 0.002 c
BL	0.551 ± 0.005 a	0.250 ± 0.007 b	0.807 ± 0.010 b	0.232 ± 0.005 a
RL	0.526 ± 0.004 a	0.604 ± 0.001 a	1.13 ± 0.010 a	0.231 ± 0.040 a
FL	0.422 ± 0.010 c	0.109 ± 0.002 d	0.531 ± 0.002 e	0.108 ± 0.001 c

**Table 3:** Effect of different light conditions on the activities of POD, SOD, and CAT antioxidant enzymes in transformed *S. bulleyana* shoots. Values marked with the same letter do not show significant differences at *p* < 0.05.

Light Condition	Enzyme Activity (U/mg of Protein)		
	POD	SOD	CAT
WL	22.26 ± 0.410 b	3.13 ± 0.440 c	2.44 ± 0.030 c
ML	12.72 ± 0.270 d	3.02 ± 0.400 c	7.80 ± 0.040 b
BL	28.56 ± 0.680 a	7.15 ± 0.400 a	8.60 ± 0.020 a
RL	14.68 ± 0.340 c	4.94 ± 0.350 b	2.34 ± 0.010 d
FL	1.49 ± 0.010 e	0.688 ± 0.230 d	0.224 ± 0.010 e

## Data Availability

The data are contained within the article.
